# Contribution of Xpert^®^ MTB/RIF to pediatric TB diagnosis in Mozambique

**DOI:** 10.5588/ijtld.22.0479

**Published:** 2023-05-01

**Authors:** C. M. Mutemba, A. Schindele, I. Munyangaju, N. Ramanlal, I. Manhiça, B. José, W. C. Buck

**Affiliations:** 1 Mozambique Ministry of Health, National Tuberculosis Control Program, Maputo, Mozambique; 2 University of Witten/Herdecke, Witten, Germany; 3 Elizabeth Glaser Pediatric AIDS Foundation, Maputo, Mozambique; 4 Barcelona Institute for Global Health (ISGlobal), Barcelona, Spain; 5 Ariel Glaser Foundation, Maputo, Mozambique; 6 University of California Los Angeles, David Geffen School of Medicine, Los Angeles, CA, USA

Dear Editor, GeneXpert testing was introduced in Mozambique in 2011. In 2013, the National TB Control Program (NTP) adopted Xpert^®^ MTB/RIF (Xpert; Cepheid, Sunnyvale, CA, USA) as the initial diagnostic test for TB in children and people living with HIV due to its higher sensitivity (compared to microscopy) and ability to identify rifampin (RIF) resistance.^1^,^2^ By 2018, there were 109 GeneXpert machines in use, and testing volume increased from 36,927 tests in 2015 to 163,530 in 2018.^3^ Sample referral networks were developed to increase access for facilities without a machine on site. During this scale-up of Xpert, the number of annual pediatric TB cases reported nearly doubled from 6,672 in 2015 to 12,522 cases in 2018. The number of bacteriologically confirmed pediatric cases also more than doubled from 677 in 2015 to 1,543 in 2018. However the percentage of cases of bacteriologically confirmed only rose from 10.1% to 12.3%, and only 60 children were treated for multidrug-resistant TB (MDR-TB) in 2018.^3^–^5^ Simultaneous to the expansion in Xpert use, there was a national training program on a new pediatric diagnostic algorithm that facilitated clinical diagnoses.^6^ This effort likely explains the large increase in overall cases, but it was unclear how Xpert expansion contributed to bacteriologic confirmation and MDR-TB treatment in children. The NTP does not receive routine programmatic data indicating the type of test done for confirmed cases, and the national GxAlert system does not provide reliable age-disaggregated information to quantify pediatric testing. Due to persistent concerns about pediatric TB incidence and case detection rates despite recent progress, we conducted a programmatic evaluation of laboratory diagnosis to estimate the contribution of Xpert to confirmed pediatric drug-susceptible and MDR-TB cases, and to evaluate age-appropriate sampling methods.^7^–^9^

Data from laboratory registers from January 2017 to December 2018 were collected from three of the 11 provinces in the country (Cabo Delgado, Gaza, Maputo Province) and compared to aggregate provincial treatment data for the same time period. All children aged 0–14 years with Xpert results registered in laboratories with a GeneXpert machine were included. This programmatic review had a non-research exemption from the National Bioethics Committee, Maputo, Mozambique (Ref 409/CNBS/19). No patient-identifying information was collected and informed consent was not required.

A total of 4,488 children were included. The median age was 8 years and the distribution of patients in the 0–4, 5–9, and 10–14-year age groups was 26.9%, 33.3% and 39.8%, respectively; 2,321 (51.7%) were female. Only 44 (1.0%) patients had documentation of previous TB treatment. The majority of specimens were respiratory, collected using spontaneous sputum production (*n* = 3,961, 88.3%), followed by sputum induction (*n* = 259, 5.8%) and gastric aspiration (*n* = 191, 4.3%). Extrapulmonary specimens, including pleural and cerebral spinal fluid, represented 1.7% (*n* = 77) of all samples. In children aged 0–4 years, respiratory specimens were obtained using sputum induction and gastric lavage in 13.5% and 14.6% of cases, respectively. Xpert results were classified as MTB-detected, MTB-not detected, and invalid in 266 (5.9%), 4,125 (91.9%) and 97 (2.2%) patients, respectively. Positivity rates were 5.0%, 5.2% and 7.1% in the 0–4, 5–9, and 10–14-year age groups, respectively. Positivity by specimen type was 6.6%, 1.9% and 1.6% for spontaneous expectorated sputum, induced sputum, and gastric aspirate specimens, respectively. Of the 266 Xpert MTB-detected samples, 11 (4.1%) were RIF-resistant, 4 (1.5%) were RIF-indeterminate, and 251 (94.4%) were RIF resistance not detected.

During the 2-year time period of the evaluation, the NTP reported 4,039 cases of TB (all forms) among children aged 0–14 years from the three provinces, including 1,161 bacteriologically confirmed cases and 26 cases of presumptive or confirmed MDR-TB.^3^ The 266 Xpert MTB-detected cases identified from the review of laboratory registers represent 6.6% of the 4,039 cases and 22.9% of the 1,161 bacteriologically confirmed cases reported. The 11 Xpert RIF-resistant cases represent 42.3% of the 26 MDR-TB cases notified (Table).^3^,^10^

**Table:** Contribution of Xpert^®^ MTB/RIF to diagnosis of DS-TB and MDR-TB in children aged 0–14 years in three provinces of Mozambique, 2017–2018.

	Cabo Delgado	Gaza	Maputo Province	Total
DS-TB cases reported	839	1,919	1,281	4,039
Xpert MTB-positive/RIF-negative or RIF-indeterminate tests	37	164	65	266
Xpert contribution to DS-TB diagnosis, %	4.4	8.5	5.1	6.6
MDR-TB cases reported	1	10	15	26
Xpert MTB-positive/RIF-positive tests	1	1	9	11
Xpert contribution to MDR-TB diagnosis, %	100	10.0	60.0	42.3

DS-TB = drug-susceptible TB; MDR-TB = multidrug-resistant TB; RIF = rifampicin.

In 2018, MDR-TB represented only 0.5% of pediatric cases reported by the NTP, and pediatric cases constituted only 5% of total cases of MDR-TB, whereas 13.4% of all reported TB cases were in children.^3^ In this evaluation, Xpert RIF-resistant tests accounted for 42.3% of reported pediatric MDR-TB cases, and we suggest that expanded access to Xpert should be prioritized to improve MDR-TB diagnosis in children, as there is limited access to TB culture and drug sensitivity testing in Mozambique.^11^

The fact that only 26.9% of Xpert tests were performed in children aged 0–4 years is worrying, as this proportion does not correlate with the known pediatric age distribution of TB disease with peak incidence in the second year of life.^12^,^13^ Based on TB incidence modeling, it is to be expected that approximately 50% of tests should be from the 0–4-year age group.^12^ However, availability of Xpert testing alone will not address this shortfall, and enhanced clinical training and mentoring for healthcare providers is needed to strengthen overall diagnostic testing of younger children. One factor likely contributing to these relatively low testing rates in younger children is difficulty with sample collection.^14^ Only 28.1% of samples in patients aged 0–4 years were recorded as induced sputum or gastric lavage, and the quality of spontaneous sputum samples is likely to be poor. Despite national efforts to scale up sputum induction through equipment distribution and training, few sites are offering this sampling modality to children with suspected TB, and gastric lavage is limited to hospitalized patients. In 2023, the NTP will launch a pilot program to use stool specimens for Xpert testing, which should help improve access for younger patients who cannot provide spontaneous sputum specimens.^15^

This evaluation had several limitations. We were not able to cross-reference laboratory registers with treatment registers, and instead used aggregate provincial treatment data, which are more prone to errors. If some patients were diagnosed but not treated, then the contribution of Xpert to notified pediatric cases may have been slightly overestimated. The laboratory data source were paper registers which also may have data quality issues, particularly for classification of specimen type, which might have led to underestimation of age-appropriate sampling methods. Furthermore, we used a convenience sampling approach that only included three of Mozambique’s 11 provinces; however, these were reasonably representative of the entire country.

In conclusion, although Xpert testing contributes substantially to detection of bacteriologically confirmed and MDR-TB cases, clinical diagnosis remains the mainstay for the diagnosis of pediatric TB in Mozambique. Surveillance and monitoring could be improved by mandatory age reporting within the GxAlert system in addition to sample type. Expansion of the GeneXpert laboratory and sample referral network, stool testing using Xpert^®^ Ultra (Cepheid) cartridges with better sensitivity, and the recent introduction of TB lipoarabinomannan testing for children living with HIV should further advance the laboratory diagnosis of drug-susceptible and drug-resistant TB in children in Mozambique.
